# Controlling proteinuria in secondary focal segmental glomerulosclerosis with cyclosporine: A case report

**DOI:** 10.1177/2050313X251353726

**Published:** 2025-07-18

**Authors:** Sohrab Kharabaf, Matthew Nguyen, Dao Le, Ramy Hanna

**Affiliations:** 1 School of Medicine, University of California, Irvine, CA, USA; 2 Division of Nephrology, Hypertension, and Kidney Transplantation, Department of Medicine, University of California, Irvine, CA, USA

**Keywords:** FSGS, cyclosporine, proteinuria, secondary glomerulosclerosis, nephrology

## Abstract

Focal segmental glomerulosclerosis is a histopathological condition characterized by podocyte injury, which manifests as persistent proteinuria and progressive decline in renal function. It is classified into primary and secondary forms, with secondary focal segmental glomerulosclerosis often resulting from factors such as obesity, hypertension, or genetic mutations. The management of secondary focal segmental glomerulosclerosis remains challenging due to the lack of standardized therapeutic guidelines. This case report describes a 21-year-old male diagnosed with biopsy-proven secondary focal segmental glomerulosclerosis, likely due to obesity-related hyperfiltration, who exhibited significant improvement in proteinuria following cyclosporine therapy. Despite initial treatment with renin–angiotensin system inhibitors and prednisone, the patient continued to have persistent proteinuria. Low-dose cyclosporine therapy was introduced, leading to a marked reduction in proteinuria without deterioration in renal function. This case highlights the potential role of cyclosporine in the management of secondary focal segmental glomerulosclerosis, particularly in cases with suspected genetic predisposition. Further research is needed to determine the long-term efficacy and safety of cyclosporine in secondary focal segmental glomerulosclerosis management.

## Introduction

Focal segmental glomerulosclerosis (FSGS) is a histopathological pattern of glomerular injury associated with significant proteinuria, leading to chronic kidney disease and end-stage renal disease.^1^ The disease can be classified into primary (idiopathic) and secondary forms. Secondary FSGS often results from maladaptive glomerular responses to conditions such as obesity, hypertension, reduced nephron mass, or genetic predisposition.^2^ The differentiation between primary and secondary FSGS is crucial, as it guides treatment strategies. While corticosteroids are the mainstay therapy for primary FSGS, secondary FSGS management focuses on addressing underlying causes and minimizing proteinuria to preserve renal function.^3^

Calcineurin inhibitors such as cyclosporine have demonstrated efficacy in reducing proteinuria in steroid-resistant FSGS, but their role in secondary FSGS remains under investigation.^4^ Given the potential for nephrotoxicity and long-term adverse effects, the use of cyclosporine in secondary FSGS must be carefully considered. Recent studies suggest that cyclosporine stabilizes podocyte structure by inhibiting calcineurin-mediated podocyte damage, which may be beneficial in select cases of secondary FSGS.^5^ Here, we present a case of biopsy-proven FSGS in a young adult, likely secondary to obesity-related hyperfiltration, successfully managed with cyclosporine.

## Case

**Figure 1.**
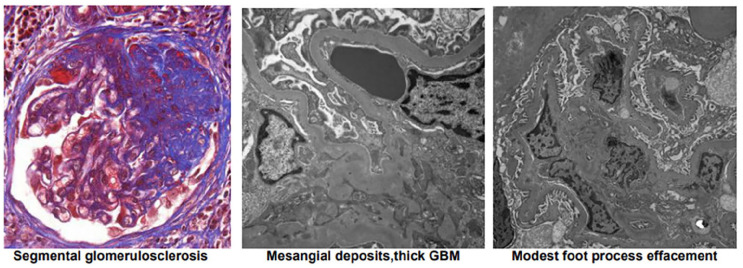
The patient’s renal biopsy showing focal and segmental glomerulosclerosis as well as mild arterial and arteriolar nephrosclerosis. The focal sclerosis is associated with small mesangial deposits and modest podocyte foot process effacement. There is very mild parenchymal scarring.

**Figure 2.**
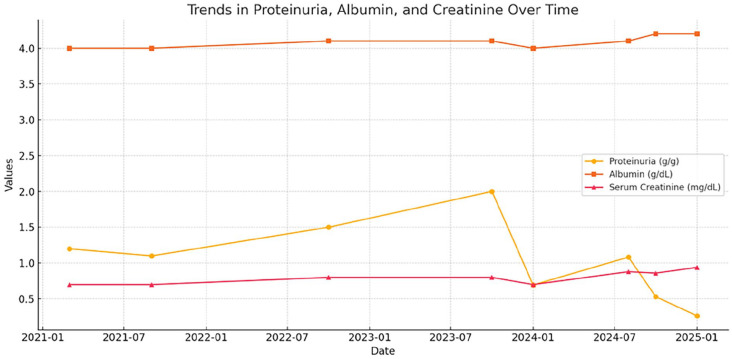
Trends in laboratory parameters over time in a patient with secondary FSGS undergoing cyclosporine therapy. Proteinuria (g/g), serum albumin (g/dL), and serum creatinine (mg/dL) were monitored across multiple clinic visits. Following the initiation and titration of cyclosporine, a marked decline in proteinuria was observed, with stable serum creatinine and albumin levels throughout the treatment period. FSGS: focal segmental glomerulosclerosis.

A 21-year-old male with a history of biopsy-proven FSGS (diagnosed at age 11) presented for nephrology follow-up. Electron microscopy demonstrated ~80% foot process effacement, consistent with podocyte injury. A representative biopsy image is included in Figure 1. He was initially managed with an angiotensin-converting enzyme (ACE) inhibitor and amlodipine for blood pressure control. His renal function remained stable with a creatinine of 0.7–0.9 mg/dL; however, persistent proteinuria (urine protein-to-creatinine ratio (UPCR) 1.0–2.0 g/g) prompted initiation of prednisone in 2019. Over the years, prednisone was gradually tapered to 1 mg daily by early 2024. Serial measurements of proteinuria, albumin, and serum creatinine were recorded before and after initiating cyclosporine therapy. A graphical plot of these values is included in Figure 2. Twenty-four-hour urine protein collections were obtained prior to (1.2 g/day) and following (0.5 g/day) cyclosporine initiation.

The patient underwent genetic testing in 2023, revealing variants of uncertain significance (VUS) in the INF2 and FN1 genes, both of which have been implicated in FSGS and proteinuria.^3^ Given ongoing proteinuria and suboptimal response to conservative therapy, cyclosporine was introduced at 25 mg twice a day and later increased to 50 mg twice a day. He demonstrated significant improvement in proteinuria, with UPCR declining from 1.0 to 0.5 g/g over several months. The initial low dose was selected to minimize nephrotoxicity, with a target trough level of 25–50 µg/L. However, the patient’s trough levels remained <25 µg/L, and dosage was empirically adjusted based on proteinuria response.

Despite the introduction of cyclosporine, the patient remained asymptomatic, with stable renal function and blood pressure well-controlled at 110–125 mmHg systolic. He was advised on lifestyle modifications, including weight management and dietary sodium restriction, and continued on enalapril 40 mg daily. Close monitoring for cyclosporine-induced nephrotoxicity and metabolic side effects was initiated, with periodic renal function assessments and cyclosporine trough level measurements. In addition, empagliflozin (an SGLT2 inhibitor) was initiated for its potential nephroprotective benefits, aligned with KDIGO 2021 recommendations.

Further follow-up demonstrated sustained reduction in proteinuria with no significant worsening of renal function. The patient’s weight remained stable, and his adherence to dietary and pharmacologic interventions was reinforced. Long-term management will focus on continued monitoring for cyclosporine nephrotoxicity, periodic reassessment of proteinuria, and further adjustments to treatment based on disease progression.

## Discussion

Secondary FSGS is a growing concern in young adults, particularly in the setting of obesity-related hyperfiltration. The pathophysiology of secondary FSGS involves glomerular hypertrophy and hyperfiltration-induced podocyte stress, leading to progressive proteinuria and sclerosis.^2^ The management of secondary FSGS typically includes renin–angiotensin–aldosterone system blockade, blood pressure control, and weight optimization to reduce glomerular stress.^4^

While corticosteroids are effective in primary FSGS, their role in secondary disease remains unclear. Calcineurin inhibitors, such as cyclosporine, have been used to stabilize podocyte function and reduce proteinuria in steroid-resistant cases, but their benefit in secondary FSGS is not well established.^5^ A landmark randomized trial by the North America Nephrotic Syndrome Study Group demonstrated that cyclosporine significantly reduced proteinuria in patients with steroid-resistant FSGS, with 70% achieving partial or complete remission compared to only 4% in the placebo group.^6^ However, relapse after discontinuation and potential nephrotoxicity were noted as concerns. This case highlights the potential for cyclosporine to mitigate proteinuria in secondary FSGS, particularly in patients with suspected genetic predisposition.

The genetic findings in this case, particularly VUS in INF2 and FN1, suggest a possible inherited component to the patient’s disease. INF2 mutations are strongly associated with both isolated FSGS and Charcot-Marie-Tooth neuropathy, while FN1 mutations have been linked to glomerulopathy with fibronectin deposits.^3^ Although these VUS are not definitively pathogenic, their presence raises the possibility of an underlying genetic predisposition that may have contributed to disease progression. Further research is needed to clarify the clinical significance of these variants and their implications for targeted therapies.

The reduction in proteinuria observed with cyclosporine therapy in this patient supports its potential role in managing select cases of secondary FSGS. However, given the risks of nephrotoxicity, careful dosing and monitoring remain essential.^4^ This case underscores the importance of individualized treatment approaches in FSGS and highlights the need for further studies to define the optimal management of secondary forms of the disease.

## Conclusion

This case highlights the potential role of cyclosporine in reducing proteinuria in secondary FSGS, particularly in cases where conservative management has been insufficient. While cyclosporine is traditionally reserved for steroid-resistant primary FSGS, its benefits in secondary FSGS, particularly in patients with possible genetic predisposition, warrant further investigation. The patient’s positive response to cyclosporine therapy supports its consideration as an adjunctive treatment in select cases of secondary FSGS.

Long-term monitoring is necessary to balance the benefits of proteinuria reduction with the risk of calcineurin inhibitor toxicity. The case underscores the importance of individualized treatment approaches in FSGS and highlights the need for future clinical studies to define optimal therapeutic strategies for secondary forms of the disease. Further research should explore the genetic underpinnings of secondary FSGS to refine treatment protocols and improve patient outcomes.
